# TNF-Alpha Inhibitors for Chronic Urticaria: Experience in 20 Patients

**DOI:** 10.1155/2013/130905

**Published:** 2013-09-18

**Authors:** Freja Lærke Sand, Simon Francis Thomsen

**Affiliations:** Department of Dermatology, Bispebjerg Hospital, University of Copenhagen, 2400 Copenhagen NV, Denmark

## Abstract

Patients with severe chronic urticaria may not respond to antihistamines, and other systemic treatment options may either be ineffective or associated with unacceptable side effects. We present data on efficacy and safety of adalimumab and etanercept in 20 adult patients with chronic urticaria. Twelve (60%) patients obtained complete or almost complete resolution of urticaria after onset of therapy with either adalimumab or etanercept. Further three patients (15%) experienced partial response. Duration of treatment ranged between 2 and 39 months. Those responding completely or almost completely had a durable response with a mean of 11 months. Six patients (30%) experienced side effects and five patients had mild recurrent upper respiratory infections, whereas one patient experienced severe CNS toxicity that could be related to treatment with TNF-alpha inhibitor. Adalimumab and etanercept may be effective and relatively safe treatment options in a significant proportion of patients with chronic urticaria who do not respond sufficiently to high-dose antihistamines or in whom standard immunosuppressive drugs are ineffective or associated with unacceptable side effects.

## 1. Introduction

Chronic urticaria not responding to high-dose antihistamines is a therapeutic challenge, and in such cases other systemic treatment options should be considered. The literature is scarce in defining effective immunosuppressive drugs that may be used for long-term treatment. Systemic corticosteroids are usually effective but are not feasible as maintenance therapy, and other immunosuppressive drugs such as azathioprine, methotrexate [1], oral tacrolimus [2], and mycophenolate mofetil [3] have only been used in case reports or small patient series. In two randomised, double-blind, and placebo-controlled trials cyclosporine A was found to be effective in controlling recalcitrant chronic urticaria [4, 5]. Finally, recent reports also point to omalizumab, a recombinant monoclonal antibody that inhibits the high-affinity Fc receptor of IgE, as an effective agent in patients with refractory chronic urticaria [6–8].

Tumour necrosis factor alpha (TNF-alpha) inhibitors have so far only been used to treat a total of eight patients with chronic urticaria according to available publications [9–11]. Here we present our experience in 20 adult patients with severe refractory chronic urticaria who were received with either adalimumab or etanercept and thereby significantly expand our knowledge of the use of TNF-alpha inhibitors for this indication.

## 2. Report

The patients described herein were a retrospective sample of patients with chronic urticaria (duration of urticaria ranged from seven months to 46 years with a mean of 13 months) seen in the outpatient clinic of a tertiary dermatological referral centre. Twenty adult patients with severe chronic urticaria with or without angioedema that was refractory to high-dose antihistamines and at least one immunosuppressive agent were offered off-label monotherapy with either adalimumab 40 mg twice monthly or etanercept 50 mg once weekly. For the main part of the patients, adalimumab was chosen over etanercept as first choice therapy, but this choice was not based on a predefined belief of superiority of this drug over the other. Previous therapy with high dose antihistamines up to four times daily of cetirizine 10 mg, loratadine 10 mg, desloratadine 5 mg, or fexofenadine 180 mg, prednisolone up to 25 mg once daily, azathioprine up to 100 mg daily, cyclosporine A up to 3 mg/kg daily, mycophenolate mofetil up to 500 mg twice daily, dapsone up to 50 mg twice daily, colchicine up to 0.5 mg twice daily, or omalizumab 300 mg once every four weeks was either ineffective or associated with unacceptable side effects, and therefore alternative therapy was considered appropriate.

Urticaria patients were screened for signs of systemic disease or chronic infection with a clinical interview, and urine analysis and culture, throat swab for streptococci, and an ice cube test for cold-induced urticaria were performed. Further evaluations were performed as appropriate including urea breath test for the diagnosis of Helicobacter pylori, stool culture, chest and sinus X-rays, and skin prick tests for common aero- or food-allergens. Blood samples were taken including complete blood count, electrolytes, thyroid stimulating hormone, antinuclear antibodies, c-reactive protein, hepatitis B and hepatitis C screening, immunoglobulins A, E, G, and M, and kidney and liver function. Furthermore, a serum-induced basophil histamine release test, HR-urticaria test, was performed (RefLab, Copenhagen, Denmark). If the HR-urticaria test was found positive (>16.5% of total histamine content), patients were categorised as having chronic autoimmune urticaria (CAU) [12]. In total, only two patients had CAU. If the HR-urticaria test was found negative (<16.5% of total histamine content), a diagnosis of chronic spontaneous urticaria (CSU) was given. CSU was diagnosed in 16 patients. One patient was diagnosed with neutrophilic urticaria (NU), whereas one patient was diagnosed with delayed pressure urticaria (DPU), respectively, based on a typical clinical and symptomatic appearance. A total of seven patients with CSU also presented with a concomitant history of angioedema (AE). 

The patients were followed up in our outpatient clinic one month after initiating therapy with TNF-alpha inhibitors, and thereafter every third month, unless side effects occurred or treatment was unsuccessful. At each visit, information about response to treatment was collected but not in a systematic manner. Based on retrospective patient records, it was possible to score the clinical response to treatment with TNF-alpha inhibitors for each patient as “complete or almost complete resolution,” “partial resolution,” (>50% reduction in symptoms and signs), and “no/limited response.” Furthermore, the duration of therapy and any signs of adverse effects of adalimumab and etanercept were recorded.

A total of 12 patients (60%) obtained resolution of urticaria and/or angioedema after initiation of therapy with either adalimumab or etanercept (Table 1). Further three patients (15%) experienced partial response to therapy. None of the patients worsened during treatment, but five patients had no benefit from the treatment. These patients were not offered treatment with another TNF-alpha inhibitor. Among responders, response to treatment was observed within the first month after initiating therapy with either adalimumab or etanercept. However, to achieve long-term relief of urticaria symptoms, continuous treatment with TNF-alpha inhibitors was needed, as intermission led to return of symptoms within a few weeks. Therefore, therapy with TNF-alpha inhibitors was not regarded as curative. The duration of treatment among responders ranged between three and 30 months with a mean of 11 months. Patients were allowed to continue antihistamines during treatment with TNF-alpha inhibitors. However, the patients who had benefit from treatment were able to reduce or discontinue antihistamines.

Six patients (30%) developed adverse reactions during treatment with adalimumab or etanercept. In five patients, these were restricted to increased frequency of mild upper respiratory infection not requiring antibiotics or hospitalization, whereas one patient, a 31-year-old female, was hospitalized due to severe headache, dizziness, vertigo, and decreased muscle strength of the upper extremities three months after initiating treatment with adalimumab. In this patient a lumbar puncture showed cerebrospinal lymphocytosis and increased protein concentration indicating a CNS toxic reaction. However, an MR scan of the cerebrum did not reveal any abnormalities and her symptoms gradually disappeared within six months following discontinuation of adalimumab.

## 3. Discussion

Among 20 patients with chronic urticaria who were unresponsive to treatment with high-dose antihistamines and one or more standard immunosuppressive therapies, we observed complete or almost complete resolution of symptoms in 60% and partial resolution in a further 15% of the patients with the TNF-alpha inhibitors adalimumab or etanercept. Responders had CSU or CAU with and without angioedema, whereas patients with DPU or NU did not respond to treatment with TNF-alpha inhibitors. Three patients discontinued treatment due to side effects. 

It is of interest that five of the 12 patients who had significant improvement with TNF-alpha inhibitors previously had failed treatment with omalizumab due to lack of efficacy or an anaphylactoid reaction. We and others have recently shown that omalizumab is effective in a significant proportion of patients with severe chronic urticaria [6–8], but this study clearly indicates that a subgroup of patients who cannot be sufficiently treated with omalizumab may obtain resolution of symptoms with a TNF-alpha inhibitor. However, it is our present position that the preferred therapy for chronic recalcitrant urticaria not responding to high-dose antihistamines and/or standard immunosuppressive drugs should be omalizumab, and that TNF-alpha inhibitors should be recommended as a second-line therapeutic option. This is based primarily on the observation that omalizumab, in comparison with TNF-alpha inhibitors for this indication, has significantly fewer adverse effects during prolonged treatment, and with a more sustained and reliable efficacy [8].

A literature search identified three publications including a total of eight patients with chronic urticaria who have received monotherapy with TNF-alpha inhibitors [9–11]. Treatment of one patient with cold urticaria [11] and one patient with DPU [9] was successful. In one small series of six patients with refractory CSU and chronic urticarial vasculitis, therapy with etanercept, adalimumab, or infliximab resulted in marked rapid improvement in all patients [10].

The pathogenesis of chronic urticaria involves mediator release, including TNF-alpha, which exists preformed in the mast cells and which is known to be newly synthesized upon mast cell activation [13]. The theoretical basis for the use of TNF-alpha targeting therapy is supported by a study that has shown that TNF-alpha is upregulated in patients with chronic urticaria compared with healthy controls [14]. In addition it has been shown that TNF-alpha is expressed throughout the epidermis in both lesional and nonlesional skin of patients with chronic urticaria but not in healthy controls [15]. Interestingly the cytokine profile in the skin of patients with chronic urticaria mimics that found in patients with psoriasis, psoriatic arthritis, and rheumatoid arthritis with increased expression of TNF-alpha and IL-10 and decreased expression of IL-2 and interferon gamma [16].

In conclusion this study suggests that TNF-alpha inhibitors could be considered in patients with severe chronic urticaria where other treatment options are contraindicated have been unsuccessful or have been associated with unacceptable adverse reactions. As this was only a small explorative cohort with no prespecified primary outcome, validated scoring system of response to treatment, or treatment algorithm for adalimumab and etanercept, it was not possible to draw any firm conclusions about superiority of one drug over the other, selective response in any urticaria subtype or with certain clinical, or paraclinical characteristics. This is a limitation of the study. Larger controlled trials with longer followup are needed to confirm the efficacy and safety of TNF-alpha inhibitors in the management of patients with severe chronic urticaria.

## Figures and Tables

**Table 1:** Characteristics of 20 patients with severe chronic urticaria treated with TNF-alpha inhibitors.

Patient characteristics			Urticaria characteristics		TNF-alpha inhibitor treatment			
No.	Sex	Age	Type	Previous treatment	Type	Duration	Effect	Side effects
1	F	43	CSU/AE	H1, Pred, and Aza	Ada	39 m	+	URI
2	F	48	CSU	H1, Pred, Aza, CyA, and MPM	Ada	30 m	++	None
3	M	64	CSU	H1, Pred, and CyA	Ada	29 m	++	None
4	F	38	CSU/AE	H1, Pred, and Oma	Ada	19 m	++	None
5	F	49	CSU	H1, CyA	Ada	16 m	+	None
6	F	31	CSU	H1, Pred, and Oma	Eta	10 m	++	None
7	F	51	NU	H1, CyA, Dap, and Col	Ada	9 m	−	None
8	M	24	CSU	H1, Pred, and Oma	Eta	8 m	++	None
9	M	58	CAU	H1, Pred, and Aza	Ada	6 m	++	None
10	F	52	CSU/AE	H1, Pred, Aza, and MPM	Ada	6 m	++	URI
11	M	29	CSU	H1, Pred, and CyA	Ada	5 m	++	None
12	F	50	CSU/AE	H1, Pred, and Oma	Eta	5 m	+	URI
13	F	74	CSU	H1, Pred, Aza, and CyA	Ada	4 m	++	None
14	F	31	CSU/AE	H1, Pred, and Oma	Ada	3 m	++	CNS
15	F	32	CAU	H1, Pred	Ada	3 m	++	URI
16	F	48	CSU	H1, Pred, and Oma	Eta	3 m	++	URI
17	F	35	CSU/AE	H1, Pred, and Aza	Ada	3 m	−	None
18	F	43	CSU/AE	H1, Pred, and CyA	Ada	3 m	−	None
19	F	48	CSU	H1, Pred, Aza, and CyA	Ada	2 m	−	None
20	M	47	DPU	H1, Aza, and Oma	Ada	2 m	−	None

CSU: chronic spontaneous urticaria; CAU: chronic autoimmune urticaria (diagnosed by serum-induced basophil histamine release test, RefLab, Copenhagen, Denmark); DPU: delayed pressure urticaria; NU: neutrophilic urticaria; AE: angioedema. H1: antihistamines; Pred: prednisolone; Aza: azathioprine; CyA: cyclosporine A; MPM: mycophenolate mofetil; Oma: omalizumab; Dap: dapsone; Col: colchicine; Ada: adalimumab; Eta: etanercept.

++: complete or almost complete response; +: partial response; −: no/limited response. URI: upper respiratory infection; CNS: CNS toxicity.
